# Modified subxiphoid approach for surgical resection of a retrosternal goiter

**DOI:** 10.3389/fsurg.2022.923389

**Published:** 2022-07-22

**Authors:** Renfeng Wang, Jianfeng Li, Jiahao Jiang, Jianyong Ding, Minghui Yang, Shuai Wang, Miao Lin

**Affiliations:** ^1^ Department of Thoracic Surgery, Zhongshan Hospital (Xiamen), Fudan University, Xiamen, China; ^2^ Department of Thoracic Surgery, Yizheng Hospital, Drum Tower Hospital Group of Nanjing, Yangzhou, China; ^3^ Department of Thoracic Surgery, Zhongshan Hospital, Fudan University, Shanghai, China; ^4^ Department of Thoracic Surgery, Taizhou Hospital of Zhejiang Province Affiliated to Wenzhou Medical University, Taizhou, China

**Keywords:** Retrosternal goiter, modified subxiphoid approach, surgical resection, VATS, surgery

## Abstract

**Backgrounds:**

Unilateral Video-Assisted Thorascopic Surgery (VATS) is a traditional minimally invasive transthoracic approach for the surgical resection of a subxiphoid goiter. Recently, the subxiphoid approach was recommended for an anterior mediastinal mass. This study aims to investigate the feasibility and efficacy of a modified subxiphoid VATS for the resection of a retrosternal goiter as an alternative transthoracic approach.

**Methods:**

We retrospectively collected all patients who underwent subxiphoid VATS for the resection of a retrosternal goiter from June 2017 to June 2021 in the Zhongshan Hospital or the Zhongshan Hospital Xiamen branch. Ten patients were found. Patient characteristics, perioperative data, and surgical information were collected and further analyzed.

**Results:**

In our study, all 10 patients underwent a thoracoscopic subxiphoid resection of a retrosternal goiter. The mean age was 49.4 years, and all were female. The majority of patients (70%) were asymptomatic. All patients were assessed by CT imaging before surgery. The mean postoperative hospital stay was 4.9 days. The drainage tube was removed 3 days after operation, and the average drainage volume was 73.1 ml. Postoperative pain was mild, with an average pain grade of 2.4 (measured on a scale from 0 to 10, with lower scores indicating less pain). There were no conversions or perioperative complications in these 10 patients.

**Conclusions:**

Most retrosternal goiters can be completely resected through the modified subxiphoid approach after an adequate preoperative evaluation and careful intraoperative management. This thoracoscopic subxiphoid approach is feasible and safe for retrosternal goiter resection.

## Introduction

A retrosternal goiter is common in anterior mediastinal masses. In 1749, Albrecht first described a retrosternal goiter and defined it as the lower margin of the mass lower than the thoracic entrance (1). According to various reports and definitions, retrosternal goiters account for about 1% to 20% of all patients with thyroidectomies (2–4). Limited by the surrounding bony structure, the mass occasionally compresses the adjacent vital organs and structures, such as the trachea, esophagus, and recurrent laryngeal nerve, causing hoarseness, dyspnea, swallowing discomfort, and other symptoms. Once diagnosed, surgery should be performed. There are many surgical approaches and strategies, such as a cervical neck incision, a combined thoracic incision, or a thoracic midline incision (5–7). With the extensive application of thoracoscopy, our research group performed a thoracoscopic resection of a retrosternal goiter using the modified subxiphoid approach and achieved good results.

## Methods

From June 2017 to June 2021, 10 patients underwent retrosternal goiter surgery in the Department of Thoracic Surgery (including Xiamen branch), Zhongshan Hospital Affiliated with Fudan University. The inclusion criteria were as follows: 50% or more of the goiter was found in the latter sternum on preoperative CT examination and postoperative pathology confirmed a thyroid mass. Data were collected, including patient history, clinical manifestations, imaging, laboratory examinations, surgical operations, postoperative pathological sections, postoperative complications, etc. Thyroid function assessment, parathyroid hormone (PTH) assessment, neck ultrasound, and chest CT were performed on all patients before surgery. A cervical color Doppler ultrasound examination of the thyroid nodule echo, calcification, boundary, and so on was used to evaluate benign and malignant statuses. The CT examination determined the size, location, and density of the retrosternal goiter, as well as its relationship to the trachea, esophagus, and surrounding large vessels. Serum calcium and parathyroid hormone levels were detected before and after the operation.

**Figure 1:**
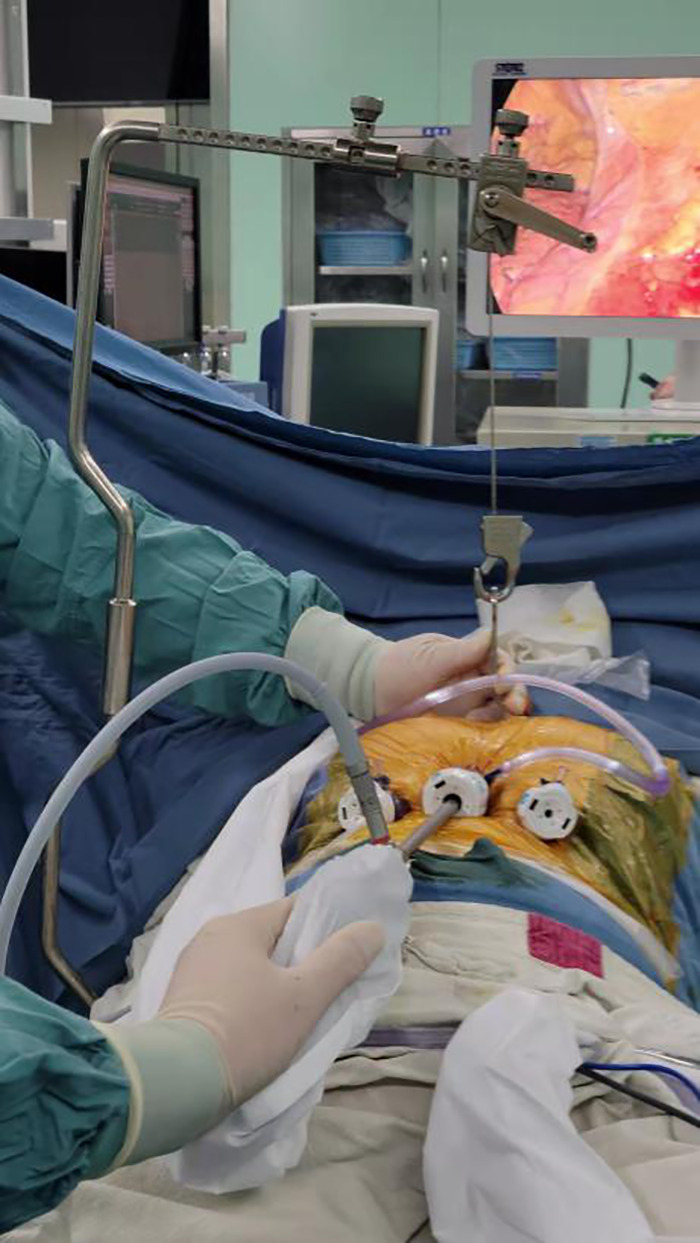
Modified subxiphoid approach for resection of a retrosternal goiter. A 10 mm trocar was placed in the subxiphoid area, and two 5 mm trocars were placed below the bilateral costal arch. A sternal retractor was used to enlarge the operative space.

**Figure 2:**
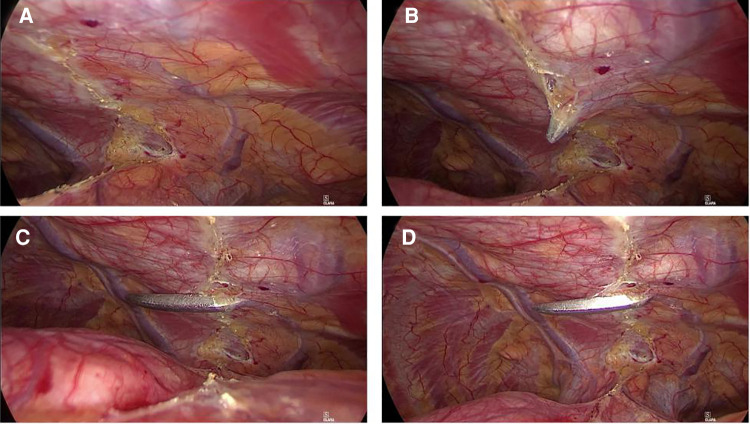
Enlarged operative space after placement of a sternal retractor. A sternal retractor was placed in the second intercostal parasternal position to elevate the sternum and enlarge the operative space.

The operation was performed in the supine scissor position as described before (8). The chest and upper abdomen were raised, and general anesthesia was performed with endotracheal intubation with a single lumen tube. Thoracotomy was prepared before the operation. Procedure: A 3-cm vertical incision was made in the subxiphoid area. After the linna alba was cut, the retrosternal space was bluntly separated, and a 10-mm trocar was placed. An endoscope was placed as an observation hole, through which CO_2_ was injected at a pressure of 8 mmHg. A 1-cm incision was made at the junction between the left and the right midclavicular line and the lower margin of the costal arch, and a 5-mm trocar was inserted as the operation hole (Figure 1). During the operation, a 1-cm incision was made between the second ribs at the left margin of the sternum, and a sternal retractor was placed (8) (Figure 2). The surgeon stood between the patient's legs. A wound drainage tube was put in place after the surgery. Postoperative changes in voice, PTH, and blood calcium were recorded.

## Results

**Figure 3:**
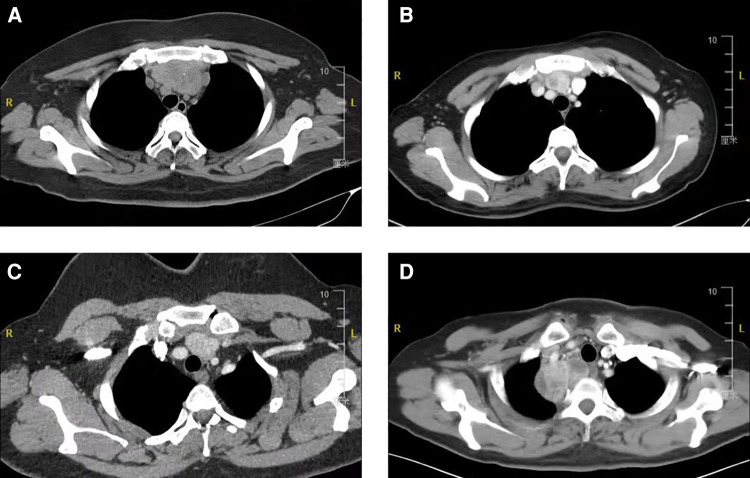
CT images of a retrosternal goiter. A series of patients with retrosternal goiters went through chest CT scan. Masses located in the mediastinum, next to the airway and great vessels, and compression on surrounding blood vessels and trachea could be observed in some cases.

**Figure 4:**
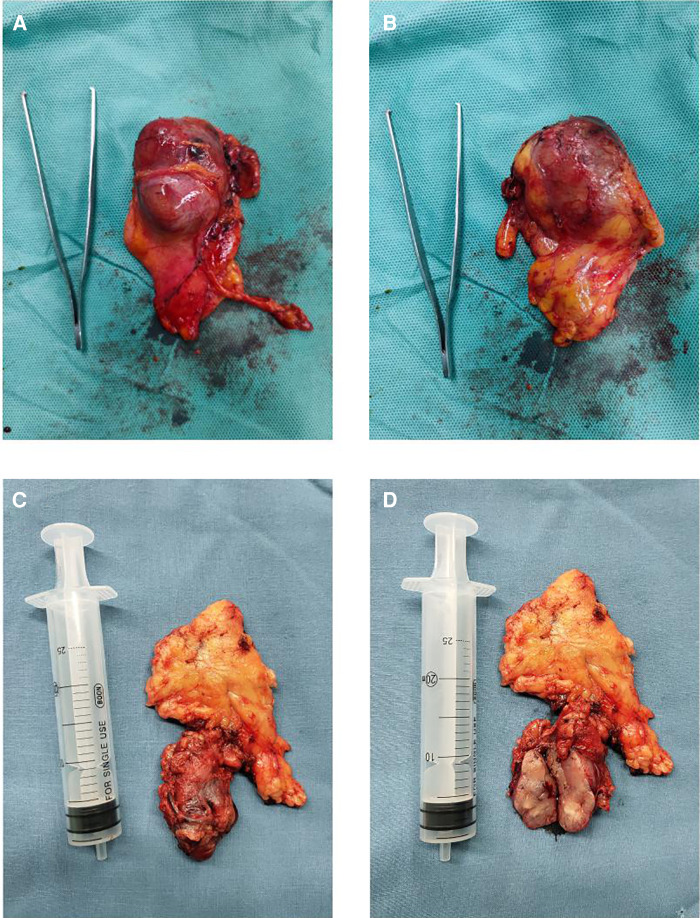
Resected goiters with surrounding tissues. Retrosternal goiters were photographed after resection. The specimen capsule is intact, and the tissue around the tumor was completely removed. Total thymectomy was performed sometimes (**C**, **D**).

**Figure 5:**
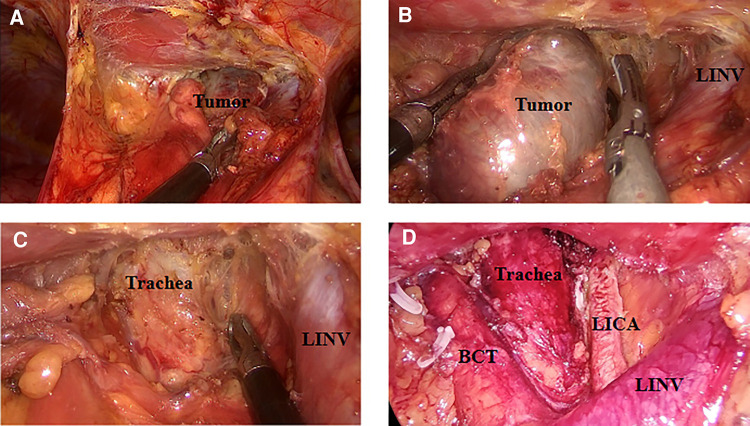
Resection of goiter through the subxiphoid approach. Mass was completely resected, and the blood vessels were clearly exposed. BCT, brachiocephalic trunk; LICA, left internal carotid artery; LINV, left innominate vein.

All 10 patients in this group were female with an onset age ranging from 34 to 72 years old (mean 49.4 years old). Of the 10 patients, 7 (70%) had no obvious symptoms, and 3 (30%) presented with discomforts, such as a neck mass, chest tightness, and chest pain. One patient (10%) had prior thyroid surgery. All patients were assessed by CT imaging before surgery (Figure 3). The maximum volume of the largest tumor was 6 × 5 cm. All 10 patients underwent the thoracoscopic modified subxiphoid approach to remove the mass, and no patient needed a neck-assisted incision or sternotomy during the surgery. R0 resection was achieved in all patients (Figures 4, 5). The mean postoperative hospital stay was 4.9 days. The average drainage volume was 73.1 ml, and the drainage tube was removed, on average, three days after surgery. Postoperative pain was mild, with an average pain grade of 2.4. No hoarseness, bleeding, wound infection, or hypocalcemia complications occurred (Table 1).

**Table 1:** Patient characteristics and postoperative complications.

Characteristics	No of patients (*n* = 10)
**Age**	49.4 (34–72)
**Sex**	
Female	10 (100%)
**Symptoms at presentation**	
Paroxysmal coughing	1 (10%)
Hyperthyroidism	1 (10%)
Dyspnea	2 (20%)
Thoracalgia	1 (10%)
Thyroid function	Normal
PTH	Normal
**Postoperative symptoms**	
Thoracic drainage (ml)	73.1 (0–320)
Postoperative pain (1–10)	2.4 (1–4)
Fever	0
Vocal cord paralysis	0
Hypocalcemia	0
Incision infection	0
Tracheomalacia	0

## Discussion

Retrosternal goiters generally grow slowly and remain asymptomatic for a long time (20%–40%). They are eventually discovered inadvertently during an examination (9–11). Some patients have symptoms of compression, such as dyspnea (12), hoarseness (13), and discomfort in swallowing (14), and although rare, some have superior vena cava syndrome or Horner syndrome (15). Surgery is the main treatment option for retrosternal goiters with or without symptoms (16). The main reasons for surgery are as follows: (I) most retrosternal goiters tend to grow and produce compression symptoms and (II) the incidence of malignant tumors is 15% (17). Therefore, even asymptomatic retrosternal goiters still require surgical treatment (15).

The best surgical approach for retrosternal goiters is still controversial. The cervical approach is recommended by many surgeons. The blood supply to a retrosternal goiter mainly comes from the inferior thyroid artery (15), but some of the blood supply might come from the aorta, innominate artery, or internal thoracic artery (18). Thus, with a simple neck incision, it is difficult to achieve accurate hemostasis. There are high risks of iatrogenic injury to the great vessels and recurrent laryngeal nerve without direct vision in the mediastinum as well. Unilateral VATS can be adopted only when the goiter is fully located on one side of the mediastinum and incidental contralateral innominate vein injury is critical. Bilateral VATS and sternotomy lead to major incisions and severe postoperative pain. Robotic-assisted surgery is also a potential choice with advantages of enhanced dexterity and greater precision (19), but further validation in retrosternal goiter resection is still needed.

Thus, we recently reported a modified subxiphoid approach as a relatively novel method for resecting an anterior mediastinum mass (8). It has also been applied for retrosternal goiter surgery by our team since 2017, and the cumulative successful cases ensured its safety and efficacy (8).

Retrosternal goiters extend below the thoracic entrance into the mediastinum (7, 17, 20, 21). Techniques for dissecting the mass are demanding due to the complex anatomy and the existence of great vessels within the narrow space. We used a sternal retractor to elevate the sternum to enlarge the surgical space and minimize the possibility of iatrogenic injury to the great vessels. Artificial pneumothorax also helps loosen the connective tissue and ease the dissection of the great vessels in the mediastinum. Goiters always have a rich blood supply. Compared with open surgery, hemostasis is more accurate with magnified vision under thoracoscopy, and the bleeding risk during surgery is reduced.

**Figure 6:**
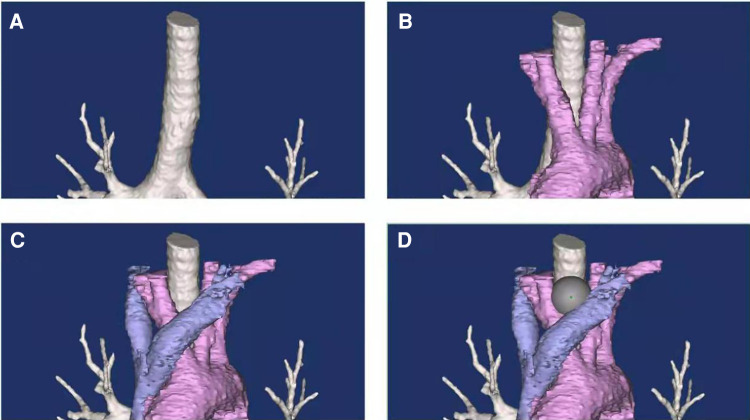
Regional anatomy in the upper mediastinum. Three-dimensional reconstruction shows the anatomy in the upper mediastinum. Innominate veins (**C**) and major arteries (**B**) could be observed. The classic retrosternal goiter locates in the niche of great vessels and the trachea (**D**).

CT scan as the gold standard for the diagnosis of retrosternal goiters (22), can evaluate the size, location, and density of a retrosternal goiter, as well as its relationship with the trachea, esophagus, and surrounding large vessels (22–24). We performed a 3D reconstruction of CT images to depict the regional anatomy intuitively (Figure 6). The location of the mass and its relationship with the adjacent structures can be observed intuitively with a modified subxiphoid approach, greatly improving the safety of the surgery. Thoracoscopy from the subxiphoid trocar provides an excellent view of the surgical area, clearly revealing the bilateral phrenic nerve and other structures that might be accidentally injured from a unilateral VATS. For example, a recurrent laryngeal nerve or phrenic nerve injury, common complications of retrosternal goiter surgeries (25, 26), did not occur in this study.

Compared with the VATS approach, the modified subxiphoid approach has been shown to have less trauma and postoperative pain (27). The surgical operation is not complex, and the learning curve is short for surgeons with VATS experience.

Our preliminary experience in intraoperative safety management to help reduce the risk of bleeding is shared here. During surgery, before dissecting the mass and vessels, it is important to place the sternal elevator, in order to enlarge the posterior sternal space for operation. Also, the surgeon should dissect the left innominate vein first and keep it in the surgical view at all times. Arteries can be found beneath the vein and mostly posterior to the mass. The dissection should be performed tightly around the mass capsular in the area near the thoracic inlet to avoid accidental vessel injury. However, in a case in which the preoperative diagnosis is incorrect, the minimally invasive surgery should be stopped and converted to open surgery if the surgeon finds the mass invading major vessels or the trachea.

## Limitations

A comparison with other approaches would help assure the non-inferior effects of the modified subxiphoid approach. However, our case volume with other approaches was too limited to perform such a comparison. We only found 3 thoracotomy cases, 3 cervical incision cases, 1 case with right VATS, and 0 cases with bilateral VATS in our database from June 2017 to June 2021. The details of these cases are presented in Supplementary Table S1.

Another limitation of our study is the small case number. One reason for this is that our team just started to do the modified subxiphoid VATS in 2017 at our center. Moreover, the incidence of retrosternal goiter is extremely low, and not all patients ask for help from thoracic surgeons.

## Conclusion

A retrosternal goiter is a relatively rare disease that presents great challenges to surgeons due to its unique anatomic location. In this study, the thoracoscopic surgery through the modified subxiphoid approach, combined with intraoperative artificial pneumothorax and sternal retractor, was performed for the resection of retrosternal goiters. Our preliminary results assure its feasibility and safety and provide surgeons with a novel alternative approach for the resection of retrosternal goiters.

## Data Availability

The original contributions presented in the study are included in the article/**Supplementary Material**, and further inquiries can be directed to the corresponding author/s.
